# Trends in Male and Female Urethral Endoscopic Management and Urethroplasty Using the TriNetX Database

**DOI:** 10.3390/jcm12062137

**Published:** 2023-03-09

**Authors:** Adam M. Ostrovsky, Zachary J. Prebay, Paul H. Chung

**Affiliations:** Department of Urology, Sidney Kimmel Medical College, Thomas Jefferson University, Philadelphia, PA 19107, USA

**Keywords:** urethral stricture, urethral stricture guidelines, urethroplasty, endoscopic treatment

## Abstract

Background: How quickly providers adapt to new practice guidelines is not well known. The objective of this study was to evaluate temporal trends in the performance of urethral endoscopic management and urethroplasty surrounding the release of the American Urological Association (AUA) Male Urethral Stricture Guidelines in 2017. We also evaluate in parallel trends in female urethral stricture disease, where AUA guidelines are not present. We hypothesized that the ratio of urethroplasty versus urethral endoscopic management in both males and females is increasing and that guidelines do not result in immediate changes in management trends. Methods: Endoscopic management and urethroplasty data were collected from the TriNetX database on adult males and females five years before (starting 1 January 2012) and after (ending 31 December 2022) the 2017 AUA guidelines. Cohorts were built using Current Procedural Terminology (CPT) codes and grouped into urethral endoscopic management (Males: CPT 52275, 52281, 52282, 53600, 53601, 53620, 53621; Females: CPT 52270, 53660, 53661, 53665) or urethroplasty (Males: CPT 53000, 53010, 53400, 53410, 53415, 53420, 53450, 53460; Females: CPT 53430). Data on patient age, race, and geographic distribution were also collected. Results: In total, 27,623 (Males: 25,039; Females: 2584) endoscopic managements and 11,771 (Males: 11,105; Females: 666) urethroplasties were reviewed across 51 Health Care Organizations. The mean age of endoscopic management and urethroplasty patients was 67.1 and 55.7, respectively (*p* < 0.01). The urethroplasty-to-endoscopic management ratio decreased for males between 2012 and 2013 and then steadily increased until 2017. The ratio steadily increased for females from 2012 to 2017. The urethroplasty-to-endoscopic management ratio showed a slight decline from 2017 to 2020 across both males and females before rising again through 2022 to a study high (Males: 0.62; Females: 0.63). Regional differences were identified, with the West having the highest urethroplasty-to-endoscopic management ratios for both males and females, the Northeast having the lowest urethroplasty-to-endoscopic management ratio for males, and the Midwest having the lowest ratio for females. Conclusions: The utilization of urethroplasty for males and females is increasing. An immediate benefit on post-guideline urethroplasty rates was not observed, and the utilization of female urethroplasty increased despite the absence of AUA guidelines. These illustrate that the impact of guideline dissemination takes time and supports the need for continued provider outreach and education on urethral stricture disease and management.

## 1. Introduction

The use of endoscopic procedures such as urethral endoscopic management to treat adult male urethral strictures is widespread. In total, 93% of urologists report using endoscopic managements to treat patients with urethral strictures [1], despite the fact that efficacy for the procedure seems to greatly drop off in the long term. The success rate of urethral endoscopic management with short strictures is around 60% at 2 to 4 years postoperatively [2], with the potential to increase stricture length over time and necessitate further procedures [3]. In contrast, open urethroplasty, a surgical procedure that has a reported success rate of over 90% [4], is less commonly used. Though urethroplasty utilization has more than doubled over the past 20 years [5], a lack of familiarity with urethroplasty and regional differences in urologic training may contribute to its lack of use despite increasing evidence of its effectiveness. Across metropolitan areas where 30 or more patients underwent repeat endoscopic management or urethroplasty, substantial variation was seen in procedure preference for stricture treatment, further highlighting the role of regional variability [6]. 

In 2017, the American Urological Association published its first set of guidelines regarding male urethral stricture disease [7]. This framework noted that urethroplasty could be offered alongside urethral endoscopic management or direct visual internal urethrotomy (DVIU) for the initial treatment of short bulbar urethral strictures (Statement 7) and that urethroplasty should be offered instead of repeated endoscopic management for recurrent anterior urethral strictures (Statement 11), among other recommendations. The impact that these guidelines had on rates of urethral endoscopic management and urethroplasty is currently unclear.

Urethral stricture disease in females is a rare diagnosis that has been inadequately understood and examined, and currently lacks a consensus as to the definition of the condition [8]. It is estimated to occur in <1 to 5% of females with lower urinary tract symptoms [9]. Like in males, treatment for female urethral stricture mostly consists of endoscopic management, with similarly poor success rates of around 43% [10]. Over the past few years, a trend towards urethroplasty to treat female urethral strictures has similarly emerged [11], though studies are often small and lack long-term follow-up data [8].

In this study, we analyzed rates of urethral endoscopic management and urethroplasty before and after the implementation of the AUA guidelines to determine whether national trends were impacted by the presence of Association recommendations. We hypothesized that both the male and female urethroplasty-to-endoscopic management ratio would increase over time and that guidelines do not result in immediate changes in management trends. Though the treatment of female urethral stricture disease was not mentioned in the AUA guidelines, the general trend towards urethroplasty, coupled with the fact that current female urethral stricture surgical treatment is largely derived from the treatment of male urethral strictures [8], prompted this hypothesis.

## 2. Methods

The data used in this study were collected between 1 October 2022 and 2 January 2023 from the TriNetX Global Collaborative Network, which provided access to electronic medical records from approximately 47 million patients across 96 healthcare organizations (HCOs) largely located in the United States. Because this study used only de-identified patient records and did not involve the collection, use, or transmittal of individually identifiable data, this study was exempt from Institutional Review Board approval. Endoscopic management and urethroplasty data were collected on adult males and females starting five years before the 2017 AUA guidelines (1 January 2012) and ending with the most current data (31 December 2022). Although the guidelines only addressed male urethral strictures, females were included to address trends across both genders.

**Figure 1:**
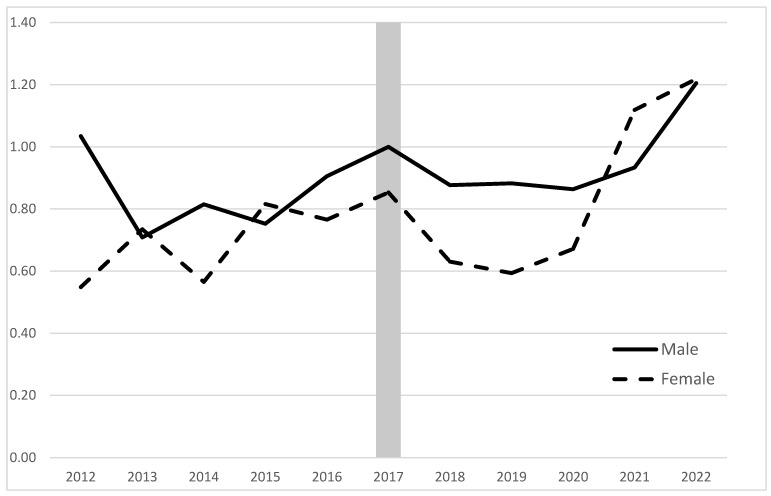
Scaled male and female ≥18 urethroplasty-to-endoscopic management ratio, adjusted for HCO number.

Urethroplasty-to-endoscopic management ratios were created for males and females, and trends were developed by year. To specifically evaluate trends over time, the 2017 male urethroplasty-to-endoscopic management ratio was standardized to 1 to ease the comparison of pre- to post-AUA guideline urethroplasty use compared to endoscopic management (Figure 1). To determine geographic distribution, patient location for both urethroplasty and endoscopic management was determined by the location of each HCO’s headquarters. Regions were divided into Northeast, Midwest, South, and West in accordance with the United States Census regions. Patients that received care in unknown or non-US HCOs had their geographic regions listed as Unknown.

Cohorts were built using Current Procedural Terminology (CPT) codes and grouped into urethral endoscopic management (Males: CPT 52275, 52281, 52282, 53600, 53601, 53620, 53621; Females: CPT 52270, 53660, 53661, 53665) or urethroplasty (Males: CPT 53000, 53010, 53400, 53410, 53415, 53420, 53450, 53460; Females: CPT 53430). A definition of each CPT code is presented in Table 1. Data on patient age, race, and geographic distribution were also collected. GraphPad Prism 9.4.0 was used for data analysis. Continuous variables were analyzed using the *t*-test and ANOVA, and categorical variables were analyzed with the chi-square test. Results were considered statistically significant at *p* < 0.05. 

## 3. Results

A total of 39,394 procedures were performed at 51 HCOs over the study period. Pre-AUA guideline publication, 9067 endoscopic managements (Males 8044; Females 1023) and 3457 urethroplasties (Males 3246; Females 211) were performed. Post-AUA guideline publication, 15,824 endoscopic managements (Males 14,521; Females 1303) and 7092 urethroplasties (Males 6703; Females 389) were performed. The mean (SD) age of endoscopic management and urethroplasty patients was 67.1 (16.7) and 55.7 (18.3), respectively (*p* < 0.01). The urethroplasty-to-endoscopic management ratio decreased for males between 2012 and 2013 (from 0.48 to 0.37) and steadily increased from 2013 to 2017 (from 0.37 to 0.52). The ratio steadily increased for females from 2012 to 2017 (0.26 to 0.44). The urethroplasty-to-endoscopic management ratio showed a slight decline from 2017 to 2020 across both males and females before rising again in 2021 and 2022 to a study-wide high (Males: 0.62; Females: 0.63). When scaled such that the 2017 male urethroplasty-to-endoscopic management ratio was standardized to 1, the male urethroplasty-to-endoscopic management ratio was seen to decrease from 1.03 in 2012 to a low of 0.71 in 2013 before steadily increasing to plateau between 0.86 and 0.93 between 2018 and 2021. The first year that the male urethroplasty-to-endoscopic management ratio increased above 1.00 (to 1.20) since 2017 was 2020. Over the same time period, the adjusted female urethroplasty-to-endoscopic management ratio vacillated between 0.55 and 0.85 between 2012 and 2020. The female ratio reached above 1.00 (1.12) for the first time in this study in 2021 before further climbing to 1.22 in 2022.

**Figure 2:**
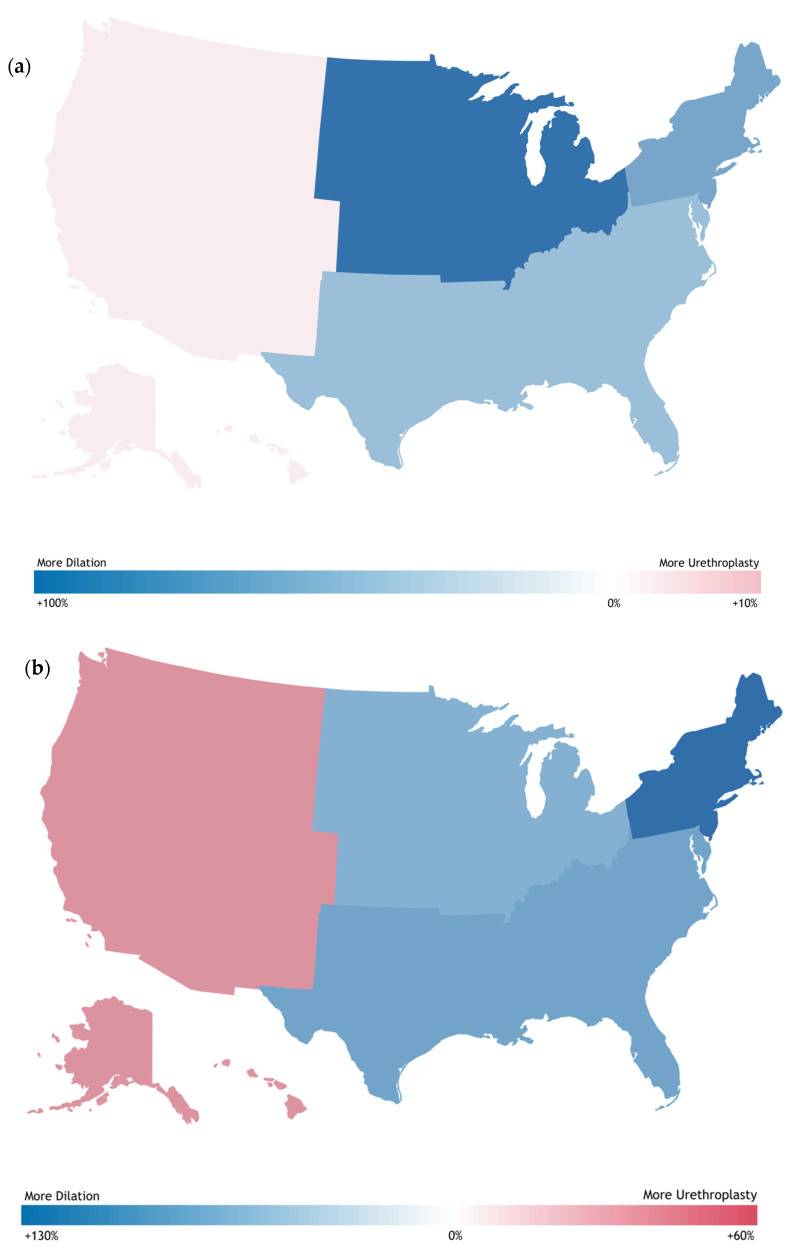
United States Maps Showing Geographic Distribution of (**a**) Male and (**b**) Female Urethroplasty–Endoscopic management Ratios in 2022 by Census Region.

Uneven geographic distribution was seen across both procedures and gender, with urethroplasties and endoscopic managements tending to be performed more in the South (47–66%) and Northeast (14–24%) and less in the West (13–19%) and Midwest (7–12%) United States (Table 2 and Table 3). United States maps demonstrating urethroplasty-to-endoscopic management ratios by census region were created for the latest year for which data was available (Figure 2), demonstrating a high of a 98.3% preference for endoscopic management over urethroplasty in the Midwest for males and a high of a 127.16% preference for endoscopic management in the Northeast for females. Urethroplasty was preferred over endoscopic management only in the West, with a 3.84% preference in males and a 57.9% preference in females. Patient race was fairly consistently represented across gender and procedure type, with patients predominantly being white (66–83%) or African American (7–19%).

## 4. Discussion

In accordance with our hypothesis, there was a modest increase in both the male and female urethroplasty-to-endoscopic management ratios across the study period. Given that the initiation of this trend preceded the AUA guidelines and that it existed across both genders, additional factors are likely to have contributed to the observed results. Further, the growth of urethroplasty immediately after 2017 was also seen to have slowed compared to pre-guideline rates, again indicating that the AUA guidelines were unlikely to be the sole driver precipitating the upward trend. When examining post-guideline trends, the impact of COVID-19 on normal practice routines also cannot be discounted. Urethroplasty surgery wait times were increased by 68% in one referral center compared to the pre-COVID-19 era [12], indicating that urethroplasty rates could continue to increase as more procedures are able to be performed.

These results are consistent with previous literature, which indicated a long-term, gradual increase in urethroplasty rates and a decrease in urethral endoscopic management rates over time rather than an abrupt increase or critical turning point [1,3]. This suggests that the introduction of the AUA guidelines served as a sign of continued support for increased urethroplasty incorporation rather than the impetus behind the change in practice patterns. However, a study examining urethroplasties and urethral dilations from 2004 to 2009 using de-identified case logs from the American Board of Urology found that 1836 urethroplasties and 19,564 urethral dilations were performed by 3877 urologists across the six years [3]. The urethroplasty-to-dilation ratio of that study, 0.09, suggests a landscape in which many more dilations are being performed than urethroplasties and is far lower than the urethroplasty-to-dilation ratio of 0.48 that we found for the first year of our study, 2012. Reconciling these numbers involves the acknowledgment that while it is impossible to know for certain which healthcare organizations are participating in TriNetX, it is likely that a large percentage are academic centers with urologists that have different experiences than general urologists. It is possible that while the AUA guidelines may influence the practice patterns of primary care urologists, referral centers had already transitioned towards performing more urethroplasties by the time of their release. This work may not reflect the practice pattern of general urologists and may conceal a greater change that could have occurred across certain subsets of urologists. Further research is needed to elucidate whether physicians across various facility types had different rates of AUA guideline adoption.

A main contributor to the increase in urethroplasties performed over time may be the increasing number of fellowship-trained reconstructive urologists who are able to offer urethroplasty more commonly. Though this may be the case, there is still room to improve. In an attempt to overcome the regional deficiencies of reconstructive urologists that may exist [3], referral to specialty, regional, or medical centers of excellence may not be a feasible or preferable option for patients. Previous work has demonstrated that 75% of patients receiving elective surgery would prefer to have the surgery at their local hospital, to the detriment of a higher mortality risk than would be afforded with travel to a larger medical center [13]. This highlights the need to continue and encourage increasing access to reconstructive urologists in all regions to ensure high-quality, equitable care for all patients.

While trends have continued to shift in favor of urethroplasty, more urethral endoscopic managements are still being performed [14]. In total, 57.8% of urologists have reported not performing urethroplasty as part of their practice, while approximately 33% have indicated that they would continue to manage long (≥3.5 cm) bulbar strictures and short (≤1 cm) bulbar strictures refractory to endoscopic management endoscopically rather than referring patients to a reconstructive specialist, despite anticipated failure [1]. 

Our work also demonstrated that nationwide, the South had the highest rates of urethroplasty, and the Midwest had the lowest rates. Previous work by Burks et al. revealed large geographic disparities in urethroplasty rates [3], which similarly continue to be present in our study. Potential factors underlying these disparities include the uneven geographic distribution of AUA residency programs, as well as differences in training and physician referral patterns. Figler et al. reported in 2014 that the presence of a reconstructive urologist in a treatment metropolitan area was associated with an increased probability of undergoing urethroplasty rather than receiving repeat endoscopic treatment (OR 2.0, 95% CI 1.7–2.5) [6]. Additionally, while a larger percentage of urethroplasty patients were treated by physicians that were members of the Society of Genitourinary Reconstructive Surgeons (76% vs. 62%, *p* < 0.001), patients receiving urethroplasty were also seen to have had to travel more to receive care, with 34% of urethroplasty patients having to travel outside of their metropolitan area for care compared to 17% for endoscopic management patients.

In examining the average ages of our cohorts, patients undergoing urethroplasty tended to be younger than those receiving endoscopic management. Levy et al. previously found that age was not an independent risk factor for urethroplasty success and that surgery suitability should rather be determined based on patients’ comorbidities and overall states of health [15]. The average male age to receive urethroplasty in our study was 55.7, which is consistent with previous work showing an evident increase in rates of urethral strictures and urethroplasties in those aged 55 and older [16]. This also demonstrates an opportunity to offer urethroplasty to more males over 60 years of age, with older age itself not acting as a deterrent to urethroplasty in support of endoscopic management. The total number of both urethroplasties and endoscopic surgeries was observed to increase across the study period. While there are a variety of factors that could be driving this growth, the advancing age of the American populace, coupled with improved surgical techniques and greater awareness among not only the general public but practicing urologists of the availability and effectiveness of these treatments, could be contributing to their increase.

The race of our patient cohorts was predominantly white (66–83%) and comparable to previous studies [5,16]; however, the percentage of African American patients (7–19%) was slightly below other reported figures. Accurate conclusions about race were challenging to draw given “Unknown” rates that reached as high as 17% and Asian and American Indian rates that were predominantly 1% or less. It has been reported in the literature that African Americans may have higher stricture rates than white Americans [16], and further research is required to account for potential racial differences and any possible correlations to higher rates of sexually transmitted diseases in African American communities [17].

Female urethral strictures represent an increasingly studied disease with complex management about which much is still not known [18]. The most common causes are idiopathic (48.5%), iatrogenic (24.1%), and traumatic (16.4%), and symptoms are often nonspecific and require a high degree of clinical suspicion to diagnose [18]. For uncomplicated female urethral strictures, it is currently acceptable to use urethral endoscopic management as a first-line treatment despite no evidence of long-term success, though urethroplasty should be pursued for patients seeking a definitive treatment for recurrent strictures [18,19]. Comparison of different urethroplasty techniques is currently not possible given that literature is mostly composed of small retrospective case series with the lack of any randomized clinical trials [8,18]. Regardless, female urethroplasty is associated with very good cure rates [19], though the optimal treatment and choice of technique still need to be elucidated in future studies.

This study has several limitations inherent to a retrospective review. Apart from relying on the accuracy and completeness of the data that was collected and entered into the registry, the TriNetX database also does not reveal the etiology of strictures or follow the stricture management history for each patient. No information on patient follow-up was available, limiting the ability to detect long-term complications or calculate success rates of different procedures. This study is also limited to patients receiving care at HCOs as part of the TriNetX network; any care received outside these HCOs would not be included. When fewer than 10 patients experience an outcome, TriNetX rounds the value to 10 to protect patient anonymity. To counteract this drawback, we ensured that none of the patient cohorts included in our study for any particular year had a sample size of fewer than 10 patients. Information about surgeon technique, experience, and stricture location and length was also not available. Additionally, referrals to urethral surgeons from primary care physicians or other urologists had the potential to mask data with respect to the geographical distribution of cases.

Still, this study was able to evaluate a large number of patients and is one of the first to evaluate urethroplasty trends in females. It is important to establish a baseline for these trends in urethroplasty and endoscopic management and how to further improve upon guideline dissemination. Furthermore, it is important to have a baseline as a new urethral dilating paclitaxel-coated balloon was recently approved by the FDA and may affect the urethroplasty–endoscopic management ratio going forward.

## 5. Conclusions

The utilization of urethroplasty for both males and females is increasing. Endoscopic management use, while still predominant, is decreasing. An immediate benefit on post-AUA guideline urethroplasty rates was not observed, and utilization of female urethroplasty increased despite the absence of AUA guidelines. These illustrate that the impact of guidelines takes time and supports the need for continued provider outreach and education on urethral stricture disease and management. Further research is needed to determine obstacles to receiving urethroplasty and how providers can best meet the needs of an increasingly aging population.

## Figures and Tables

**Table 1:** Common Procedural Terminology Codes and Definitions.

CPT Code	Definition
52270	Cysto w/internal urethrotomy, female
52275	Cysto w/internal urethrotomy, male
52281	Cysto w/calibration and dilation urethral stricture
52282	Cysto insertion of perm urethral stent
53000	Urethrotomy/Urethrostomy, pendulous urethra
53010	Urethrotomy/Urethrostomy, perineal urethra
53400	Urethroplasty, first stage for fistula, or stricture
53410	Urethroplasty, 1-stage reconstruction of male anterior urethra
53415	Urethroplasty, transpubic or perineal, 1-stage, for membranous/prostatic urethra
53420	Urethroplasty, two-stage reconstruction of membranous/prostatic urethra, first stage
53430	Urethroplasty, reconstruction of female urethra
53450	Urethromeatoplasty, with mucosal advancement
53460	Urethromeatoplasty, with partial excision of distal urethral segment
53600	Dilation of male urethra, initial
53601	Dilation of male urethra, subsequent
53620	Dilation of urethral stricture by passage of filiform and followers, initial
53621	Dilation of urethral stricture by passage of filiform and followers, subsequent
53660	Dilation of female urethra, initial
53661	Dilation of female urethra, subsequent
53665	Dilation of female urethra, under anesthesia

**Table 2:** Comparison of Male ≥ 18 Urethroplasty and Endoscopic management Procedures Centered Around 2017 Guideline Changes.

	Endoscopic Management		Urethroplasties	
	2012–2016	2018–2022	2012–2016	2018–2022
n	8044	14,521	3246	6703
Age, Mean (SD)	71 (17)	67 (16)	43 (26)	40 (27)
Race (%)				
White	74	75	71	66
African American	13	12	15	14
Asian	2	2	1	2
American Indian	0	0	0	1
Unknown	11	11	13	17
Geographic Distribution (%)				
Northeast	34	27	21	21
Midwest	19	12	12	8
South	33	42	51	47
West	14	14	15	19
Unknown	0	4	<1	5

**Table 3:** Comparison of Female ≥ 18 Urethroplasty and Endoscopic management Procedures Centered Around 2017 Guideline Changes.

	Endoscopic Management		Urethroplasties	
	2012–2016	2018–2022	2012–2016	2018–2022
n	1023	1303	211	389
Age, Mean (SD)	72 (15)	67 (15)	60 (17)	52 (17)
Race (%)				
White	83	80	67	69
African American	7	10	18	19
Asian	1	1	4	3
American Indian	0	1	4	2
Unknown	8	8	11	7
Geographic Distribution (%)				
Northeast	16	34	14	24
Midwest	24	12	7	10
South	56	46	66	47
West	4	6	13	17
Unknown	0	1	0	2

## Data Availability

Data proprietary to TriNetX.
